# A pH-Responsive Hydrogel for the Oral Delivery of Ursolic Acid: A Pentacyclic Triterpenoid Phytochemical

**DOI:** 10.3390/gels10090602

**Published:** 2024-09-22

**Authors:** Carlos D. Gutierrez, Rosana L. Aranzábal, Ana M. Lechuga, Carlos A. Serrano, Flor Meza, Carlos Elvira, Alberto Gallardo, Michael A. Ludeña

**Affiliations:** 1 Department of Chemistry, Faculty of Chemical, Physical and Mathematical Sciences, National University of San Antonio Abad del Cusco (UNSAAC), Av. De la Cultura 733, Cusco 921, Peru; 170595@unsaac.edu.pe (C.D.G.); rosana.aranzabal@unsaac.edu.pe (R.L.A.); ana.lechuga@unsaac.edu.pe (A.M.L.); carlos.serrano@unsaac.edu.pe (C.A.S.); 2 Technology of Materials for Environmental Remediation Group (TecMARA), Faculty of Sciences, National University of Engineering, Av. Tupac Amaru 210, Rimac 15333, Peru; flor.meza.l@uni.pe; 3 Institute of Polymer Science and Technology (ICTP-CSIC), Juan de la Cierva 3, 28006 Madrid, Spain; celvira@ictp.csic.es (C.E.); gallardo@ictp.csic.es (A.G.)

**Keywords:** hydrogel, pH-responsive, ursolic acid, release, delivery, itaconic acid, 2-hydroxyethylmethacrylate

## Abstract

In this study, poly(HEMA-PEGxMEM-IA) hydrogels were prepared by radical copolymerization of poly(ethylene glycol) methyl ether methacrylate (PEGxMEM), 2-hydroxyethyl methacrylate (HEMA), and itaconic acid (IA). The reaction was carried out in ethanolic solution using N,N′-methylenebisacrylamide (MBA) as a crosslinking agent and 1-hydroxycyclohexyl phenyl ketone (HCPK) as a photo-initiator. The poly(HEMA-PEGxMEM-IA) hydrogels (HGx) were evaluated as a delivery system for ursolic acid (UA), a phytochemical extracted from the plant *Clinopodium revolutum,* “flor de arena”. The hydrogels were characterized by Fourier-transform infrared spectroscopy (FTIR-ATR), Raman spectroscopy, X-Ray diffraction (XRD), thermogravimetric analysis (TGA), and scanning electron microscopy (SEM). The swelling behavior was studied in buffer solutions from pH 2 to 10, specifically at pH 2.2 (gastric environment) and 7.4 (intestinal environment). It was found that the hydrogels studied showed sensitivity to pH. At pH 2.2, the degree of swelling for HG_5_ and HG_9_ hydrogels was 0.45 and 0.93 (g water/g hydrogel), respectively. At pH 7.4, the degree of swelling for HG_5_ and HG_9_ hydrogels was 1.97 and 2.64 (g water/g hydrogel), respectively. The SEM images show the variation in pore size as a function of pH, and the UA crystals in the pores of the hydrogels can also be observed. The in vitro UA release data best fit the Korsmeyer–Peppas kinetic model and the diffusion exponent indicates that the release mechanism is governed by Fickian diffusion.

## 1. Introduction

**Figure 1:**
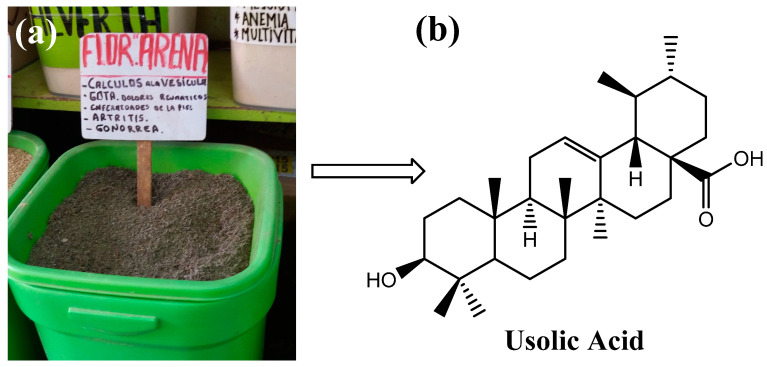
Commercialization of flor de arena in herb shops in Peru (**a**) and structure of ursolic acid (**b**).

Ursolic acid (UA) is a pentacyclic triterpene of pharmacological interest due to its various bioactive properties such as antioxidant, anti-inflammatory [1], antimicrobial [2], and anti-cancer properties [3]. This compound can be isolated from several plants such as *Rosmarinus officinalis*, *Malus domestica*, *Origanum vulgare*, *Salvia officinalis* [4], and *Clinopodium revolutum*, an endemic Peruvian plant commonly called “flor de arena” [5] (Figure 1). Due to the poor solubility of UA in water, the molecule has a low bioavailability, which hinders its clinical application. For this reason, studies in recent years have focused on the development of systems that transport UA to the site of absorption to improve its bioavailability [6,7,8]. Among these delivery systems, hydrogels are a promising approach because these polymeric materials can respond to external stimuli [9,10,11]. In particular, pH-responsive hydrogels have shown great potential as oral delivery systems for bioactive compounds because they respond to changes in the pH value of the gastrointestinal tract. This means that hydrogels can be designed to retain a bioactive agent under certain conditions (e.g., in the stomach) and release it under other conditions (e.g., in the intestine) [12]. For this reason, pH-sensitive hydrogels can deliver the compound to the site of absorption and release its cargo without causing side effects [13].

Hydrogels based on poly(2-hydroxyethylmethacrylate) poly(HEMA) are of special interest because they have been used in a wide range of biomedical applications, such as contact lenses, tissue regeneration, drug delivery, wound dressings, etc. [14]. However, it is necessary to incorporate monomers with ionizable groups such as –COOH or –SO_3_H to prepare pH-responsive hydrogels. Itaconic acid (IA) is an excellent choice because it has two carboxylic groups and is obtained from natural sources [15,16]. On the other hand, hydrogels based on poly(ethylene glycol) methyl methacrylate (PEGxMEM) have shown good biocompatibility, which means that they do not cause any adverse effects when in contact with any part of the human body [17,18,19,20].

The objective of this study was to prepare a hydrogel capable of transporting UA to the site of absorption (intestine) in order to increase its bioavailability and to take advantage of its full pharmacological potential. For this purpose, hydrogels based on poly(HEMA-PEGxMEM-IA) loaded with UA were prepared. The polymerization was performed by radical light curing. The hydrogels were instrumentally characterized by FTIR-ATR, RAMAN, XRD, and scanning electron microscopy (SEM). In addition, the degree of swelling of the hydrogels in water was evaluated, as well as their response to pH changes, simulating the physiological conditions of the human body. Finally, an in vitro UA release study was carried out.

Swelling analysis and SEM images showed that the hydrogels are pH sensitive, which is very useful for the delivery of active substances to a specific site such as the intestine.

## 2. Results and Discussion

**Figure 2:**
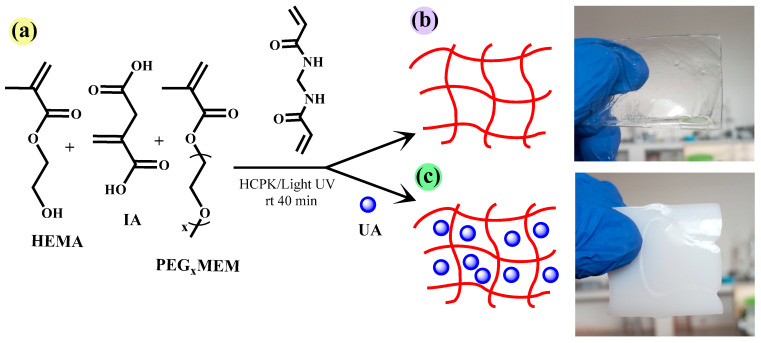
Monomers and crosslinking agents used to prepare the hydrogels (**a**), schematic representation of the hydrogel HG (**b**), and hydrogel loaded with UA in situ HGx-UA (**c**).

In order to transport UA to the adsorption site and improve the bioavailability of this molecule, we selected the monomers HEMA, PEG_x_MEM, and IA to prepare hydrogels (Figure 2b,c). Free radical polymerization was initiated by HCPK, and was photo-activated. These free radicals propagate with the HEMA, PEGxMEM, and IA monomers to form polymer chains. The crosslinking agent MBA facilitates the formation of chemical bonds between the polymer chains, creating a three-dimensional network (Figure 2a).

### 2.1. Characterizations

**Figure 3:**
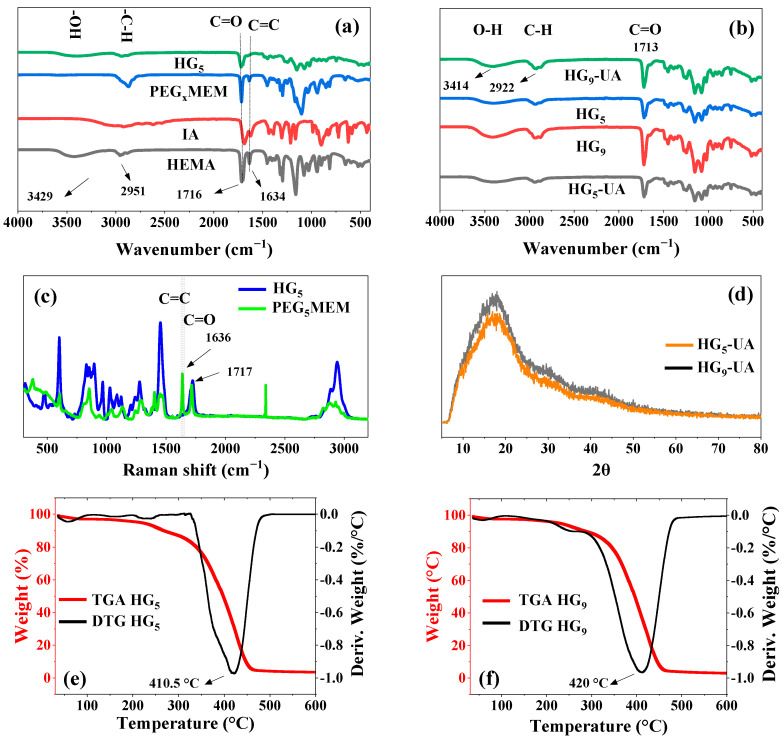
FT-IR spectra of HG_5_ and monomers (**a**), FT-IR spectra of hydrogels (HG_5_ and HG_9_) and hydrogels loaded with UA (HG_5_-UA and HG_9_-UA) (**b**), Raman spectrum of PEG_5_MEM and HG_5_ (**c**), XRD analysis of HG_5_-UA and HG_9_-UA (**d**), TGA/DTG curves of HG_5_ (**e**), and TGA/DTG curves of HG_9_ (**f**).

FT-IR spectroscopy was used to confirm the chemical composition of the prepared hydrogels (Figure 3a). Characteristic signals are observed in the spectra of the monomers HEMA, PEG_5_MEM, IA, and the HG_5_ hydrogel. HEMA shows peaks at 3429 cm^−1^ (–OH), between 3000 cm^−1^ and 2800 cm^−1^ (–CH, –CH_2_, CH_3_), 1716 cm^−1^ (C=O), and 1634 cm^−1^ (C=C) [21,22]. The IA monomer shows a broad band between 3230 cm^−1^ and 2750 cm^−1^ (–OH, of carboxylic acid), 2922 cm^−1^ (C–H), and 1689 cm^−1^ (C=O) [23]. In PEG_5_MEM, the signals at 2873 cm^−1^, 1689 cm^−1^, 1634 cm^−1^, and 1100 cm^−1^ correspond to C–H, C=O, C=C, and C–O–C groups, respectively [24]. The spectrum of the HG_5_ hydrogel has peaks that are characteristic of the HEMA, PEG_5_MEM, and IA monomers, except the C=C peak, indicating that these groups carry out the polymerization. Figure 3b shows the FT-IR spectra of the HGx and HGx-UA hydrogels; the characteristic UA peaks at 3420 cm^−1^ (–OH), 2975 to 2950 cm^−1^ (–CH, –CH_2_, –CH_3_), 1714 cm^−1^ (C=O), and 1680 cm^−1^ (C=C) are overlapped by the hydrogel signals.

RAMAN and FT-IR are complementary techniques that provide detailed information about vibrational modes. The C=C vibrations are particularly intense and well defined in RAMAN spectra [25]. For this reason, an analysis of HG_5_ was carried out and compared with that of the monomer PEG_5_MEM (Figure 3c). Figure 3c shows the characteristic vibrations of PEG_5_MEM; the peak at 1636 cm^−1^ corresponds to the C=C group, which is absent in the HG_5_ spectrum, indicating a successful polymerization through these groups and a good purification of the hydrogel to remove unreacted monomers trapped in the hydrogel matrix.

The XRD patterns of UA are found at angles of 2θ = 10.76, 14.15, 16.49, 21.9, 26.4, and 40.22 [26,27]. XRD analysis was performed for HG_5_-UA and HG_9_-UA hydrogels to observe the UA peaks. However, due to the amorphous nature of the hydrogels, only a broadband of 2θ = 10 to 25 was observed overlapping the crystallographic patterns of the UA crystals (Figure 3d).

The thermal stability of the HGx hydrogels was analyzed by TGA (Figure 3e,f). The TGA curves show three main stages of weight loss. The first stage of weight loss, reaching 100 °C, could be associated with water loss [28]. The second slight weight loss around 300 °C could be attributed to water loss generated by the ester formation through the carboxyl groups (–COOH) of the IA and the hydroxyl group (–OH) of the HEMA monomers [15]. The third stage corresponds to the thermal degradation of the hydrogels; the maximum degradation rate occurs at 410.5 °C and 429 °C for hydrogel HG_5_ and HG_9_, respectively. Since the hydrogels are intended to be used as UA transport systems, which is carried out at physiological temperature (37 °C), the prepared hydrogels will be thermally stable in this environment.

### 2.2. Swelling Behavior (q)

**Figure 4:**
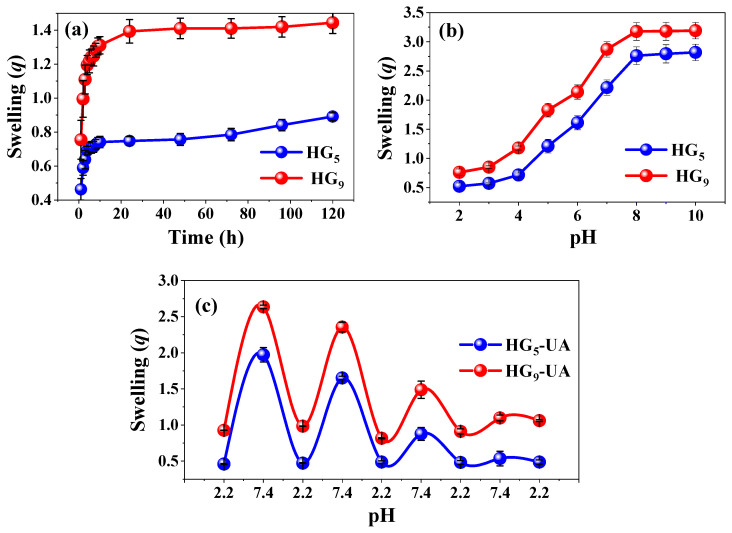
Swelling behavior in water (**a**), pH medium (**b**), and cyclic pH response (**c**).

The swelling behavior of HG_5_ and HG_9_ hydrogels in water was investigated. The degree of swelling influences the mechanical properties and the mobilization of the bioactive compound through the polymeric network [29]. Figure 4a shows the results; HG_5_ and HG_9_ have a *q_max_* of 0.89 and 1.44 (g H_2_O/g hydrogel), respectively. The degree of swelling is influenced by the inter/intramolecular hydrogen bonds between IA-IA (–COOH·····HOOC–) and IA-HEMA (–COOH·····HO–), which may be more favored in shorter polymer chains, restricting the relaxation of the polymer chains and causing a slight decrease in water absorption. In longer chains such as PEG_9_MEM, there is a lower tendency to form inter/intramolecular hydrogen bonds between IA-IA (–COOH–HOOC–) and IA-HEMA (–COOH·····HO–), but a higher tendency to form IA-H_2_O (–COOH·····HO–) and HEMA-H_2_O (–OH·····HO–) bonds [15,30,31].

The pH response of HG_5_ and HG_9_ hydrogels was evaluated from pH 2 to 10 at 37 °C. The swelling degree is related to the amount of ionizable carboxylic groups in the polymer network. Figure 4b shows that both HG_5_ and HG_9_ hydrogels respond to pH due to the dissociation of the IA carboxylic groups pK_1_ = 3.85 and pK_2_ = 5.45. The carboxylic groups remain protonated (–COOH) at pH values below their dissociation constants, resulting in low swelling values. However, at pH values above their dissociation constants, the carboxylic groups tend to deprotonate (–COO^−^), becoming negatively charged and causing an electrostatic repulsion that favors the expansion of the hydrogel [32,33].

Cyclic swelling of HG_5_-UA and HG_9_-UA hydrogels was evaluated in buffer solutions simulating gastrointestinal conditions: pH 2.2 and 7.4 at 37 °C (Figure 4c). Figure 4c shows that in the first cycle, HG_5_-UA reaches a *q_max_* of 0.45 at pH 2.2 and 1.97 at pH 7.4. In the case of HG_9_-UA, the *q_max_* is 0.93 at pH 2.2 and 2.64 at pH 7.4. A decrease in swelling capacity after each cycle is noted, attributable to ion–dipole interactions between polymer chains [23].

### 2.3. SEM Characterization

**Figure 5:**
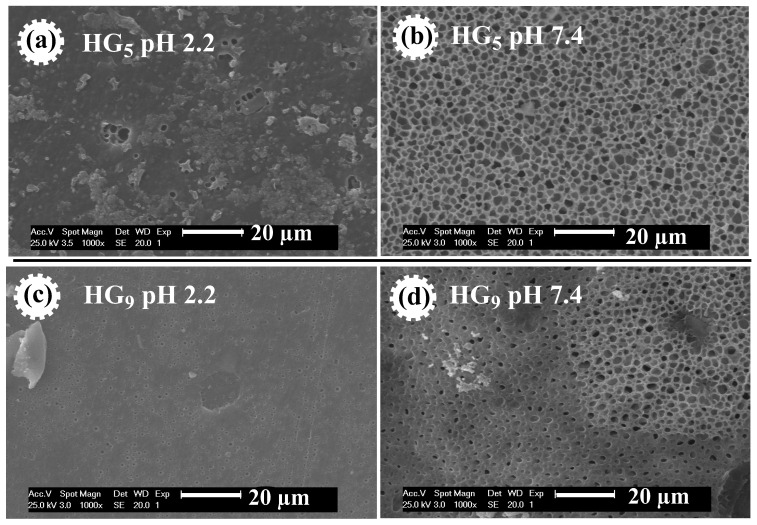
SEM micrographs of HG_5_ hydrogel at pH 2.2 (**a**) and at pH 7.4 (**b**), and of HG_9_ hydrogel at pH 2.2 (**c**) and at pH 7.4 (**d**).

Figure 5 shows the morphology of HG_5_ and HG_9_ hydrogels. The effect of pH on pore size is observed in both hydrogels. At pH 2.2, the pores of the hydrogels are closed, because at acidic pH levels the carboxylic groups (–COOH) of the IA are protonated and form hydrogel bonds that prevent the expansion of the hydrogel. At pH 7.4, open and evenly distributed pores are observed because at basic pH levels the carboxyl groups of the IA deprotonate (–COO^−^), resulting in electrostatic repulse between the polymer chains, causing them to swell and expand. The porosity favors better water retention, loading, and diffusion of ursolic acid through the polymer matrix. These results are consistent with the values obtained for the swelling at different pH values since porous materials favor greater water penetration through the polymeric networks [34].

**Figure 6:**
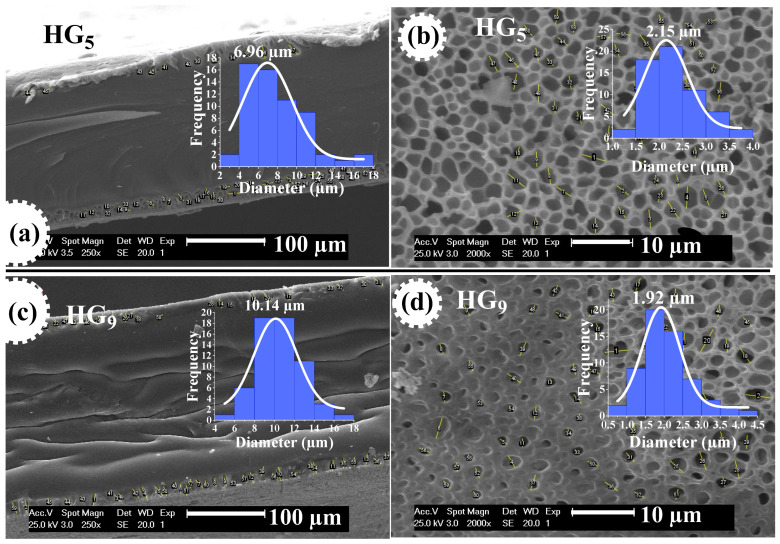
The pore size of HG_5_ hydrogel at contour (**a**) and surface (**b**), and of HG_9_ hydrogel at contour (**c**) and surface (**d**), both at pH 7.4.

Figure 6 shows SEM micrographs of HG_5_ and HG_9_ hydrogels at pH 7.4, where the pore size measurement performed on the hydrogel outline shows an average value of 6.96 and 10.14 μm, respectively. This could be attributed to the pore depth of the hydrogels. The pore size on the surface of HG_5_ and HG_9_ hydrogels is 2.15 and 1.92 μm, respectively. The larger pore depth in HG_9_ compared to HG_5_ also explains the higher degree of swelling of the HG_9_ hydrogel.

**Figure 7:**
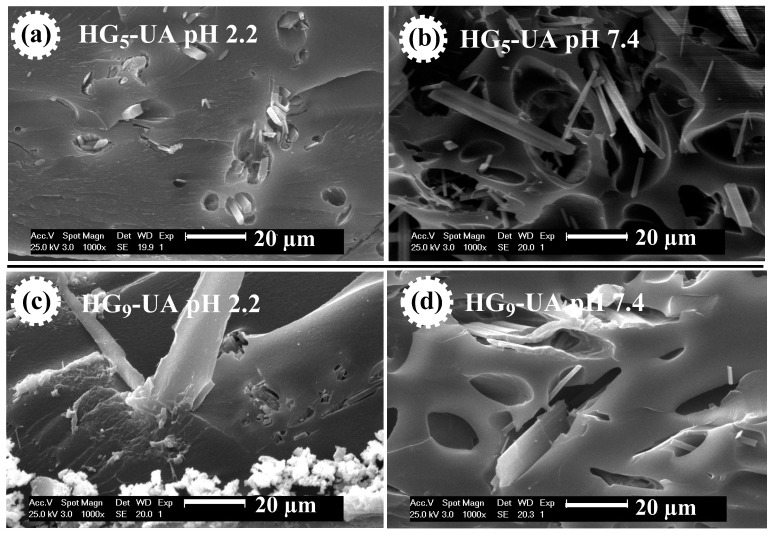
SEM micrographs of HG_5_-UA hydrogel at pH 2.2 (**a**) and at pH 7.4 (**b**) and of HG_9_-UA hydrogel at pH 2.2 (**c**) and at pH 7.4 (**d**).

SEM micrographs were also taken of the UA-loaded hydrogels, i.e., HG_5_-UA and HG_9_-UA, at pH 2.2 and 7.4 (Figure 7). Figure 7 shows the UA crystals in the pores of the hydrogels. At pH 2.2 the pores are compressed, trapping the UA crystals, which would prevent the release of UA in an acidic environment. At pH 7.4 the pores are open, which would allow the release of UA in a basic environment.

### 2.4. Determination of Drug Loading and Encapsulation Efficiency

Table 1 shows the drug loading content and encapsulation efficiency of the HG_5_-UA and HG_9_-UA hydrogels. These DLC and EE values are comparable to those reported by Das et al. [35], who achieved 3.35% loading and 55.52% encapsulation. Other authors such as Antonio et al. [36] achieved 97.47% encapsulation; Payomhom et al. [37] achieved 1.5% loading and 25% encapsulation. It has been observed that by increasing the amount of UA, the system does not gel. Attempts were made to load HG_5_ and HG_9_ hydrogels with UA by adsorption, but low DLC (1.45% and 1.38%) and EE (2.89% and 2.76%) values were obtained, respectively. Although experiments were also performed with solvents such as ethanol and methanol, in which UA is more soluble, low DLC and EE values were obtained. For this reason, the remaining experiments were carried out with in situ loaded hydrogel, i.e., HG_5_-UA and HG_9_-UA.

### 2.5. In Vitro Release of HG_5_-UA and HG_9_-UA

**Figure 8:**
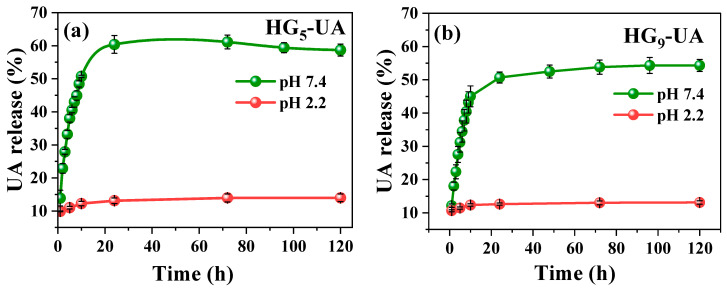
UA release profiles of HG_5_-UA (**a**) and HG_9_-UA (**b**) hydrogels at pH 2.2 and 7.4.

The in vitro release of UA from hydrogels was evaluated in buffer pH 2.2 (acidic environment of the stomach) and buffer pH 7.4 (basic environment of the intestine) with 3% Tween 80 at 37 °C [35]. For the application of the hydrogel as a transport system for hydrophobic bioactive compounds such as UA in the intestine, it is necessary that the hydrogel does not release its charge in an acidic medium and has a good release in a basic medium. From this point of view, the HG_5_-UA and HG_9_-UA hydrogels have the desired release profile (Figure 8a,b). In buffer pH 2.2, the release of UA from HG_5_-UA and HG_9_-UA is low due to the low degree of swelling of the hydrogels at acidic pH levels. In buffer pH 7.4, a higher amount was released due to the higher diffusion of UA from the swollen pores in basic media. Analyzing the UA release profiles for the two hydrogels, the release increases rapidly at the beginning and gradually reaches the equilibrium value in approximately 24 h. Figure 8 also shows that the percentage release with a PEG_5_MEM monomer is slightly higher than with PEG_9_MEM. This can be explained by the chain length as PEG_9_MEM is a longer chain monomer; the UA is trapped in the matrix and diffusion is more difficult. This also explains the incomplete release of UA from the hydrogels.

In order to establish the mechanism of UA release from the HG_5_-UA and HG_9_-UA hydrogels, the data obtained from the in vitro release were fitted to different kinetic models: zero-order, first-order, Korsmeyer–Peppas, Hixson–Crowell, and Higuchi [38]. The calculated parameters are presented in Table 2 and the sum of squared residuals (SSR), Akaike Information Criterion (AIC), and the coefficient of determination (R²) were evaluated to identify the model that best fits the release data. The SSR, which quantifies the error between the experimental data and the values predicted by the model, is lower for the Korsmeyer–Peppas model (0.582 for HG_5_-UA and 0.578 for HG_9_-UA). The AIC, which balances goodness of fit with model simplicity, also showed the lowest values for the Korsmeyer–Peppas model (−40.527 for HG_5_-UA and −40.619 for HG_9_-UA). Furthermore, the R^2^ values were 0.754 for HG_5_-UA and 0.783 for HG_9_-UA, indicating that the Korsmeyer–Peppas model *M_t_/M_e_ = kt^n^* best describes the UA release mechanism from the hydrogels.

The value of the exponent “*n*” in the Korsmeyer–Peppas model is associated with the type of transport mechanism. Values of *n* ≤ 0.45 indicate a predominantly Fickian diffusion-controlled release. Values of *n* close to 1 indicate a release controlled by polymer chain relaxation, corresponding to a zero-order kinetic model. Intermediate *n* values between 0.45 and 1 indicate anomalous transport where both diffusion and relaxation influence the release, which can be studied using non-Fickian diffusion [38,39]. In this study, the *n* values obtained were 0.256 and 0.278 for HG_5_-UA and HG_9_-UA, respectively, suggesting a quasi-Fickian diffusion release. This behavior is attributed to diffusion controlled mainly by the movement of the bioactive compound through the polymer matrix, which relaxes and swells in a basic environment (pH 7.4) [40].

## 3. Conclusions

To improve the oral bioavailability of hydrophobic phytochemicals, an approach based on the use of sensitive hydrogels is presented. The hydrophobic phytochemical UA was loaded in situ into the pH-sensitive poly(HEMA-PEG_x_MEM-IA) hydrogels with an efficiency of more than 85%. From the observations of buffer swelling (pH 2.2 and 7.4) and SEM images, the hydrogels can protect UA in the stomach, where the pH is close to 2.2, and release its cargo in the intestine, where the pH is close to 7.4. The results obtained from the in vitro release under conditions simulating the gastrointestinal environment fit the Korsmeyer–Peppas kinetic model, suggesting a mechanism of diffusion through the hydrogel. Finally, this study provides relevant information for developing targeted transport systems for oral delivery of hydrophobic phytochemicals to exploit the full pharmacological potential of these molecules.

## 4. Materials and Methods

### 4.1. Materials

The main materials used for the synthesis of the hydrogel in this study were the monomers 2-hydroxyethyl methacrylate (HEMA, 97%), polyethylene glycol methyl ether methacrylate (PEG_5_MEM, Mn average 300), polyethylene glycol methyl ether methacrylate (PEG_9_MEM, Mn average 500), itaconic acid (IA, ≥ 99%), crosslinker N,N′-methylenebisacrylamide (MBA, 99%), initiator 1-hydroxycyclohexyl phenyl ketone (HCPK, 99%), and TWEEN 80. These materials were purchased from Sigma-Aldrich (St. Louis, MO, USA).

### 4.2. Extraction of Ursolic Acid

**Figure 9:**
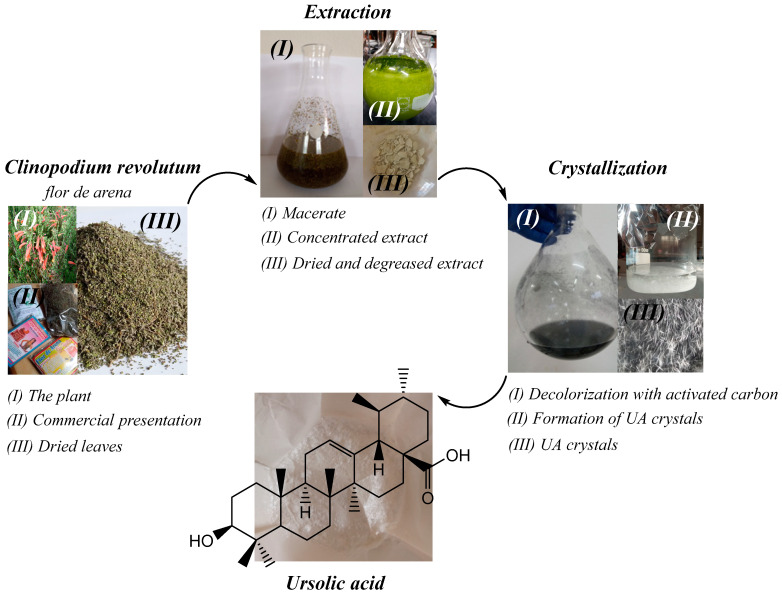
Process of extraction of UA from *Clinopodium revolutum,* “flor de arena”.

Ursolic acid (UA) was extracted from the medicinal plant *Clinopodium revolutum*, which is known as flor de arena in local herbal shops. The methodology was developed in a previous work [5]; 50 g of dried leaves of *Clinopodium revolutum* was macerated in 200 mL of ethanol (96%) for 24 h. Then, it was filtered and macerated again. The process was repeated three times. The ethanolic extract obtained was concentrated to dryness using a rotary evaporator, dried, and degreased with petroleum ether. The degreased extract was redissolved in 150 mL of ethanol (96%) and kept refrigerated for 24 h. The precipitate obtained was filtered and dried in an oven at 60 °C for 12 h. Crystallization was carried out in 90 mL of ethanol (96%) at 60 °C and 5 g of activated carbon was used for decolorization. The mixture was kept under constant stirring for 5 min and then filtered with a vacuum pump. The filtrate obtained was crystallized at 4 °C for 12 h. After this time, the crystals were filtered, washed with cold ethanol, and dried in an oven at 60 °C for 1 h. The extraction procedure is shown in Figure 9.

The obtained UA crystals were analyzed by (a) UV-VIS (Thermo Scientific, Genesys 150, Waltham, MA, USA): The sample was dissolved in methanol and scanned from 200 to 750 nm. The spectrum showed a single absorption band at 210 nm, characteristic of this compound. (b) HPLC (Thermo Scientific, Ultimate 3000Thermo, Sunnyvale, CA, USA): For this analysis, a column Phenomenex RP-C18 250 × 4.6 mm and 5 μm was used. Analysis conditions were mobile phase (water/acetonitrile 2:8), flow rate 1 mL/min, isocratic, 5 µL injection, and detection at 210 nm. The chromatogram shows a peak at rt 11.30 min with a relative area of 97.52%, which corresponds to the percentage purity of the UA. (c) FTIR (Thermo, Nicolet 380, Waltham, MA, USA): The FTIR-ATR spectrum of UA crystals previously dried at 60 °C for 1 h shows characteristic peaks of this molecule at 3420 cm^−1^ (–OH), 2975 to 2950 cm^−1^ (–CH, –CH_2_, –CH_3_), 1714 cm^−1^ (C=O), and 1680 cm^−1^ (C=C).

An exhaustive characterization is not necessary, as it has been shown that only UA can be obtained from *Clinopodium revolutum* using the described method [5,41].

### 4.3. Synthesis of Hydrogels HG_x_-UA

The synthesis of hydrogels based on poly(HEMA-PEGxMEM-IA) loaded in situ with UA (HGx-UA) was performed following the methodology proposed by Gallardo et al. [42], with some modifications. The monomers IA, HEMA, PEGxMEM, crosslinker MBA, and UA were mixed with 300 µL of ethanol under stirring at 40 °C for 5 min. The mixture was then bubbled with nitrogen gas for 10 min and the initiator HCPK was added. The solution was immediately injected into a 53 × 26 × 2 mm mold and placed in the photopolymerization chamber (4 watts, 365 nm) for 40 min. After this time, the hydrogels were removed from the mold and cut. Purification was performed by immersing the hydrogels in distilled water, which was renewed several times to remove unreacted substances. Finally, the hydrogels were dried at room temperature. Blank hydrogels (HG_x_) were prepared similarly without the addition of UA. The feed compositions used to synthesize the hydrogels are shown in Table 3.

### 4.4. Characterization

The FT-IR spectra were obtained using an FT-IR spectrometer (Perkin Elmer, Spectrum TWO, S/N 117248, Waltham, MA, USA). The samples were dried and crushed before being placed on the ATR crystal. Measurements were taken at room temperature over the range of wave numbers from 4000 to 400 cm^−1^. A RAMAN instrument (Renishaw InVia Raman Microscope, S/N 9W596, Wotton-under-Edge, Gloucestershire, UK) was used as a complementary technique to the FTIR-ATR analysis. The hydrogels loaded with UA were analyzed using X-Ray diffraction (XRD-BRUKER D8-ADVANCE S/N 203691 SAP 604310, Karlsruhe, Germany, Cu, λ = 1.5406 Ǻ). The hydrogels were previously dried and ground, and the analysis was performed as 2θ = 5°–80°. Thermogravimetric analysis (TGA) of the hydrogels was performed on a Perkin Elmer STA6000 (Waltham, MA, USA) instrument, using 10 mg of previously dried and ground hydrogels. The heating ramp was from 37 °C to 600 °C at a heating rate of 5 °C/min in an inert nitrogen gas atmosphere at a flow rate of 20 mL/min. The morphology of HG_x_ and HG_x_-UA hydrogels was observed by scanning electron microscopy (SEM, XL30ESEM Philips, North Billerica, MA, USA). The hydrogels were previously swollen in pH 2.2 and pH 7.4 buffer at 37 °C for 24 h and then freeze-dried for 12 h. Metallization with Au/Pd alloy (80/20) was required for better observation.

### 4.5. Swelling Behavior (q)

The degree of hydrogel swelling (*q*) was determined using Equation (1). For this purpose, 20 mg of dried hydrogel (*W_d_*) was weighed, immersed in 30 mL of water, and allowed to swell. Then, it was removed from the liquid and weighed (*W_s_*); it is necessary to dry the surface of the hydrogel before weighing. The degree of swelling was determined every 1 h for the first 10 h, then every 24 h. This study was performed in triplicate.

(1) $$q=\frac{W_{s}-W_{d}}{W_{d}}$$

The pH response was evaluated by determining the degree of swelling in various buffer solutions (pH 2 to 10) at 37 °C. 

### 4.6. Drug Loading and Encapsulation Efficiency

The drug loading and encapsulation efficiency of UA were determined by a UV-VIS spectrophotometer. To determine the amount of UA entrapped in the hydrogel, the samples were first disrupted by immersing the crushed hydrogel in methanol for 24 h and then sonicated for 1 h. Briefly, 20 mg of HG_5_-UA and HG_9_-UA were crushed and immersed in methanol (5 mL) for 24 h, then sonicated for 1 h and their absorbance was determined at 210 nm. A calibration curve at 210 nm was previously performed with a methanol solution of UA from 5 µg/mL to 100 µg/mL.

The drug loading content (DLC) and encapsulation efficiency (EE) of ursolic acid were determined by Equations (2) and (3), respectively [35].

(2) $$Drug\;loading\;content\;(\%)=\frac{Weight\;of\;UA\;in\;hydrogel\;}{weight\;of\;hydrogel}\times 100$$

(3) $$Encapsulation\;efficiency\;(\%)=\frac{Weight\;of\;UA\;in\;hydrogel\;}{Initial\;amount\;of\;UA}\times 100$$

### 4.7. In Vitro Drug Release Study

In vitro release of UA from the HG_x_-UA hydrogels was evaluated by immersion in buffer with Tween 80 [35]. A total of 20 mg of HG_x_-UA was immersed in 5 mL of 0.1 M phosphate-buffered saline (PBS, pH 7.4) containing Tween 80 (3% *v*/*v*) at 37 °C with gentle agitation. At a predetermined time (1 h, 2 h, 3 h, 4 h, 5 h, 6 h, 7 h, 8 h, 9 h, 10 h, 24 h, 48 h, and 72 h), the hydrogels were removed and the surface was washed with water. The hydrogel was then disrupted by immersion in 5 mL of methanol for 24 h and sonication for 1 h. The absorbance of the methanol solution was recorded at 210 nm and the concentration of UA remaining in the hydrogel was determined. The amount of UA released is equal to the amount of initial UA minus the amount of UA remaining in the hydrogel.

The percentage of release was determined using Equation (4) [43].

(4) $$\%\;release=\frac{W_{t}}{W_{0}}\times 100$$

where *W_t_* is the amount of UA released at time t and *W_0_* is the amount of initial hydrogel UA. The same procedure was carried out with a buffer of pH 2.2.

## Figures and Tables

**Table 1:** Drug loading content (DLC) and encapsulation efficiency (EE) of hydrogels.

Hydrogel	Initial UA (mg)	After Purification UA (mg)	Mass HGx (g)	DLC (%)	EE (%)
HG_5_-UA	35.00	30.34	0.947	3.20	86.69
HG_9_-UA	35.00	30.05	1.119	2.69	85.86

**Table 2:** Parameters calculated by fitting release data using various kinetic models.

HG_5_-UA					
Model	C_o_	k	SSR	AIC	R^2^
zero-order	36.446	0.253	1490.330	69.348	0.454
first-order	6.018	0.002	0.079	−68.548	0.520
Hixson–Crowell	-	0.006	0.709	−37.755	0.499
Higuchi	-	3.569	1089.188	64.958	0.618
Korsmeyer–Peppas	-	22.340	0.582	−40.527	0.754
HG_9_-UA					
Model	C_o_	k	SSR	AIC	R^2^
zero-order	30.946	0.252	1226.977	66.626	0.509
first-order	6.263	0.002	0.050	−74.927	0.584
Hixson–Crowell	-	0.006	0.487	−43.018	0.559
Higuchi	-	3.475	833.657	61.215	0.667
Korsmeyer–Peppas	-	18.359	0.578	−40.619	0.783

**Table 3:** Feed compositions of the poly(HEMA-PEG_x_MEM-IA) hydrogels (HG_x_).

Hydrogel	HEMA g (mol %)	PEG_5_MEM g (mol %)	PEG_9_MEM g (mol %)	IA g (mol %)	UA g	MBA g	HCPK g
HG_5_-UA	0.800 (80%)	0.230 (10%)	-	0.100 (10%)	0.035	0.023	0.0156
HG_9_-UA	0.800 (80%)	-	0.384 (10%)	0.100 (10%)	0.035	0.023	0.0156

## Data Availability

The original contributions presented in the study are included in the article, further inquiries can be directed to the corresponding author.
